# Milk and wheat allergy, and celiac disease

**DOI:** 10.1186/2045-7022-1-S1-S37

**Published:** 2011-08-12

**Authors:** Mika Mäkelä

**Affiliations:** 1 Helsinki University Central Hospital, Department of Allergy , Skin and Allergy Hospital, Helsinki, Finland

## 

Cow's milk, egg, wheat, soy, peanut, and fish account for about 90% of the food allergies but there are considerable differences from one country to another. Milk allergy is among the best characterized food allergies, particularly in infants and small children. Only part of the children with early allergy to cow's milk grow into shool-age with milk allergy but those that do often have severe symptoms and high specific IgE level to milk. In accordance, attaining tolerance to cow's milk is associated with decreased epitope binding by IgE and a concurrent increase in corresponding epitope binding by IgG4. Recent data point that analysis of IgE response to milk components may help in determining those with higher risk for persistent or more severe milk allergy.

Among wheat allergic patients, co-sensitization to cow's milk and hen's egg is common. Wheat ingestion may trigger immunoglobulin E (IgE)-mediated immediate symptoms, including urticaria, angioedema, bronchial obstruction, nausea and abdominal pain, or in severe cases, even systemic anaphylaxis. Delayed reactions include gastrointestinal symptoms and worsen-ing of atopic dermatitis. In addition to food allergy, wheat ingestion may cause exercise-induced anaphylaxis, or celiac disease (CD), and inhalation and handling of wheat flour aller-gens may lead to respiratory allergy, or contact urticaria on the skin.

Wheat IgE is commonly found in all ages of atopic children when whole grains are used as an antigen either in skin prick tests or serum IgE measurements. The specificity of this IgE re-sponse in identifying clinical disease, however, is poor. It is of importance to recognize dis-ease-determining components in the IgE response. Several studies have shown that sensiti-zation particularly to omega-5 gliadin is associated with challenge-proven wheat-allergy. Moreover, recent data imply that gliadin-positive patients have more likely severe reactions after wheat ingestion. A well-characterized severe clinical entity is also wheat-dependent, ex-ercise-induced anaphylaxis in which sensitization to omega-5 gliadin is a prerequisite.

We have analyzed especially the gliadin-sepcific responses in wheat allergy. Sensitization to gliadin with a SPT wheal of ≥5 mm at the time of the diagnostic challenge was associated with a slower course of recovery from wheat hypersensitivity (p = 0.019), and a SPT wheal of ≥ 3 mm to gliadin at any time was associated with the development of asthma. Recently, we examined IgE response to 22 wheat components in children with challenge positive wheat allergy and their controls. The results show that wheat-allergy diagnosis can be improved considerably by component-based ap-proach. In the future, wheat IgE diagnosis should include at least measurement of IgE to gli-adins.

Gluten and its major component, gliadin, is essential also in the pathogenesis of celiac dis-ease. Gliadin can be a target of both cellular and humoral immune responses. Approximately 90 percent of patients with celiac disease have the HLA-DQ2 haplotype, explaining the family-association of the disease but still leaving open the wide variability in prevalence between dif-ferent Caucasian populations. Both IgA and IgG antibodies can be seen in CD patients but the most sensitive diagnostic work-up is to define tissue transglutaminase (tTG) transglutami-nase IgA antibodies.

